# Phosphatidylserine Decarboxylase Promotes Ferroptosis Through STAT3/GPX4 Signaling in Gastric Cancer

**DOI:** 10.3390/cimb48030300

**Published:** 2026-03-11

**Authors:** Li Wang, Yaoxing Wang, Mingkai Shao, Tao Wang, Wanbao Zheng, Jun Cao, Renwen Luo, Youyan Tu, Yiting Xia, Yiming Wei, Ning Liu, Wenjie Lu, Youzhi Xu

**Affiliations:** 1 College of Basic Medicine, Anhui Medical University, Hefei 230032, China; 2345010251@stu.ahmu.edu.cn (L.W.); wyx18473449151@126.com (Y.W.); 2345010097@stu.ahmu.edu.cn (M.S.); 2313100046@stu.ahmu.edu.cn (Y.X.); w18726870301@163.com (Y.W.); 2 Inspection Department, Yuetang District Center for Disease Control and Prevention, Xiangtan 411100, China; 3 School of Pharmacy, Anhui Medical University, Hefei 230032, China; 2345010146@stu.ahmu.edu.cn (T.W.); 2345010176@stu.ahmu.edu.cn (W.Z.); caojun20201314@163.com (J.C.); 2445010199@stu.ahmu.edu.cn (R.L.); 2445010150@stu.ahmu.edu.cn (Y.T.); liunky@163.com (N.L.); 4 School of Life Sciences, Westlake University, Hangzhou 310000, China

**Keywords:** gastric cancer, mitochondria, phosphatidylserine decarboxylase, phosphatidylethanolamine, ferroptosis, STAT3, GPX4

## Abstract

Gastric cancer (GC) remains a major global health burden, and increasing evidence suggests that ferroptosis plays an important role in regulating tumor cell survival. Phosphatidylserine decarboxylase (PISD) is a key mitochondrial enzyme responsible for phosphatidylethanolamine (PE) synthesis; however, its molecular function in GC remains poorly understood. In this study, we suggest that downregulation of PISD is associated with enhanced ferroptosis in GC cells by disrupting mitochondrial PE homeostasis and impairing mitochondrial function. Mechanistically, PISD depletion reduces PE levels, is accompanied by a reduction in signal transducer and activator of transcription 3 (STAT3) phosphorylation, and decreases GPX4 expression, leading to enhanced lipid peroxidation, iron accumulation, and redox imbalance. Pharmacological inhibition of ferroptosis using Ferrostatin-1 (Fer-1), activation of STAT3 by ML115, or supplementation with lysophosphatidylethanolamine (LPE) partially rescues PISD knockdown-induced ferroptosis. In vivo, PISD downregulation is significantly accompanied by a reduction in tumor growth in GC xenograft models. Collectively, our findings reveal a previously unrecognized role of PISD in linking mitochondrial phospholipid metabolism to STAT3/GPX4-dependent ferroptosis, providing mechanistic insights into the regulation of ferroptosis in gastric cancer.

## 1. Introduction

GC is one of the most common malignancies worldwide, with poor clinical outcomes and a 5-year survival rate of 20% [1]. Despite its declining incidence in certain regions, GC remains the third leading cause of cancer-related mortality [2,3,4]. Risk factors include Helicobacter pylori infection, high salt intake, aging, and poor dietary habits [5,6]. Late diagnosis and insufficient stratification of patients by molecular subtype further worsen the prognosis [7,8,9]. With advances in genomic technologies, the identification of novel molecular regulators is urgently needed [10].

PISD is required for maintaining mitochondrial PE synthesis [11,12,13]. Deficiency of mitochondrial PE disrupts energy metabolism, inflammatory responses, and cancer cell behavior [14,15,16,17,18]. Mitochondria, the cellular energy centers, dynamically adapt energy production to meet the cell’s requirements and also play a pivotal role in the proinflammatory response and combating pathogenic infections [19]. The biochemical processes occurring within the mitochondria of cancer cells and the stromal cells that interact with them are required for maintaining tumor metastasis [20]. Prior studies suggest that PISD acts as a regulator of tumor-initiating cells, and its overexpression is accompanied by a reduction in breast cancer metastasis [21]. Conversely, PISD downregulation delays autophagy [22]. However, its function in GC remains unclear.

Ferroptosis is a unique form of iron- and lipid-peroxidation-dependent cell death [23,24], distinct from apoptosis, necrosis, and autophagy. It plays a crucial role in suppressing GC proliferation, metastasis, and chemoresistance [25,26,27,28]. Among its regulators, GPX4 serves as a negative modulator that detoxifies lipid peroxides [29].

In this study, we show that reduced PISD expression decreases PE levels, impairs mitochondrial function, and inhibits STAT3/GPX4 signaling, leading to ferroptosis in GC cells. These findings provide mechanistic insights into the role of PISD in regulating mitochondrial function and ferroptosis in gastric cancer.

## 2. Materials and Methods

### 2.1. Cells and Cell Culture

Human gastric cancer cell lines (AGS, MKN45, and MGC-803) were obtained from the Shanghai Institute of Biochemistry and Cell Biology, Chinese Academy of Sciences (Shanghai, China). AGS and MKN45 were cultured in RPMI-1640 medium (Gibco, Grand Island, NY, USA), and MGC-803 cells were cultured in DMEM (Gibco, Grand Island, NY, USA), supplemented with 10% fetal bovine serum (FBS; Gibco, Grand Island, NY, USA) and 100 U/mL penicillin and 100 μg/mL streptomycin. All the cells were incubated in a CO_2_ incubator (5% CO_2_, 37 °C).

### 2.2. Immunofluorescence (IF) Staining

Sterile glass coverslips were placed in 24-well plates and coated with poly-L-lysine (Sigma-Aldrich, St. Louis, MO, USA) for 15 min, followed by three washes with phosphate-buffered saline (PBS). AGS and MKN45 cells were seeded onto the coated coverslips and subjected to the indicated treatments. After treatment, cells were fixed with 4% paraformaldehyde for 10 min at room temperature and washed three times with PBS. Cells were permeabilized with 1% Triton X-100 in PBS for 10 min and blocked with 5% bovine serum albumin (Sigma-Aldrich, St. Louis, MO, USA) for 1 h at room temperature. Cells were incubated with the indicated primary antibodies overnight at 4 °C. After three washes with PBS, cells were incubated with Cy3-conjugated goat anti-rabbit IgG (H + L) secondary antibody (1:100; Proteintech, Wuhan, China) for 1 h at room temperature in the dark. Nuclei were counterstained with DAPI (1:10,000) for 10 min. Coverslips were carefully removed from the 24-well plates and mounted onto glass slides using an antifade mounting medium (Fluoromount-G, Southern Biotech, Birmingham, AL, USA), allowed to cure, and sealed with nail polish prior to imaging. Confocal images were acquired using a Nikon A1R/A1 laser-scanning confocal microscope (Nikon, Tokyo, Japan) under identical laser power, gain, and exposure settings for all groups. Imaging was performed on glass-mounted samples to avoid autofluorescence from plastic culture plates. Negative controls without primary antibody were included to evaluate background fluorescence. Fluorescence intensity was quantified using ImageJ software (version 1.53q; National Institutes of Health, Bethesda, MD, USA). All experiments were independently repeated at least three times.

### 2.3. Western Blotting

The cells were washed with PBS, incubated with RIPA lysis buffer (containing protease inhibitors) (Beyotime, Shanghai, China), and lysed on ice for 10 min. The lysate was centrifuged at 12,000× *g* at 4 °C for 20 min. A BCA protein determination kit (Beyotime, Shanghai, China) was used to measure the protein concentration. Proteins were separated by 10% SDS-PAGE and transferred to polyvinylidene difluoride membranes (Millipore, Burlington, MA, USA), which were blocked with 5% nonfat milk for 2 h. The membranes were incubated overnight at 4 °C with the following specific antibodies: anti-PISD (Santa Cruz Biotechnology, Dallas, TX, USA; CAT# sc-390070 and Proteintech, Wuhan, China; CAT# 68484-1-Ig), anti-STAT3 (Proteintech, Wuhan, China; CAT# 60199-1-Ig), anti-pSTAT3 (Proteintech, Wuhan, China; Cat No. 60479-1-Ig), anti-GPX4 (Zenoio, Chengdu, China; CAT# R24461), anti-GAPDH (Affinity Biosciences, Cincinnati, OH, USA; CAT# T0004), anti-JAK2 (Servicebio, Wuhan, China; CAT# GB11325-50), and anti-pJAK2 (Servicebio, Wuhan, China; CAT# GB114585-100). After washing with TBST three times, the membranes were incubated with an HRP-conjugated secondary antibody at room temperature for 1 h. Finally, the membranes were washed and exposed to an ECL substrate (GE Healthcare, Shanghai, China). Signals were visualized with an enhanced chemiluminescence instrument (Thermo Fisher Scientific, Waltham, MA, USA). The intensity of the bands was quantified using ImageJ software.

### 2.4. Lipid Peroxidation MDA Assay Kit

Intracellular malondialdehyde (MDA) levels were measured using an MDA assay kit (ABBKINE, Wuhan, China) according to the manufacturer’s instructions. Briefly, treated cells grown in six-well plates were harvested, lysed in IP lysis buffer, and centrifuged at 12,000× *g* for 10 min at 4 °C. The protein concentrations in the supernatants were determined using a BCA Protein Assay Kit (Beyotime, Shanghai, China). Equal amounts of protein were mixed with the MDA working solution and incubated in a boiling water bath (100 °C) for 15 min to allow for the formation of the MDA–thiobarbituric acid (TBA) adduct. After cooling to room temperature, the mixtures were centrifuged at 1000× *g* for 10 min. Finally, 200 μL of each supernatant was transferred to a 96-well plate, and absorbance was measured at 532 nm using a microplate reader (Thermo Fisher Scientific, Waltham, MA, USA). MDA concentrations were calculated from a standard curve, normalized to protein content, and expressed as nmol/mg protein.

### 2.5. Cell Ferrous Iron (Fe^2+^) Fluorometric Assay Kit

Fe^2+^ levels were measured using a Fe^2+^ Fluorometric Assay Kit (Elabscience, Wuhan, China), according to the manufacturer’s instructions. Briefly, treated cells cultured in six-well plates were harvested and lysed in lysis buffer. The lysates were centrifuged at 12,000× *g* for 10 min at 4 °C, and the supernatants were collected for analysis. Equal volumes of sample supernatant were mixed with the working reagents supplied with the kit and incubated at room temperature in the dark for the time specified in the manual to allow the formation of the Fe^2+^–chromogenic complex. After incubation, 200 μL of each reaction mixture was transferred to a 96-well plate, and the absorbance was measured at the recommended wavelength (typically around 542 nm) using a microplate reader (Thermo Fisher Scientific, Waltham, MA, USA). Fe^2+^ concentrations were calculated from a standard iron calibration curve and normalized to the cellular protein content or cell number.

### 2.6. Glutathione (GSH) Assay Kit

GSH levels were measured using a GSH Assay Kit (Nanjing Jiancheng Bioengineering Institute, Nanjing, China) according to the manufacturer’s instructions. Briefly, the treated cells cultured in six-well plates were harvested, washed twice with cold PBS, and lysed in the recommended lysis buffer. The lysates were centrifuged at 12,000× *g* for 10 min at 4 °C, and the supernatants were collected for analysis. Equal volumes of sample supernatant and the kit’s working reagents were mixed and incubated at room temperature for the time specified in the kit protocol to enable the formation of the yellow-colored 5-thio-2-nitrobenzoic acid (TNB) product. Absorbance was measured at 405 nm using a microplate reader (Thermo Fisher Scientific, Waltham, MA, USA). GSH concentrations were calculated from a standard calibration curve and normalized to the cellular protein content or cell number.

### 2.7. Cell Proliferation and Colony Formation Assays

Cell proliferation was assessed using the CCK-8 cell counting kit (Dojindo Laboratories, Kumamoto, Japan). The cells were seeded in 96-well plates at a density of 3 × 10^3^ cells/well. Five replicates were used for each group. The cells were treated with LPE (25 μM) or Fer-1 (2 μM) for 48 h to assess cell proliferation. Next, the cells were treated with 10 μL of CCK-8 reagent, added to each well in the dark, and then incubated for 2 h at 37 °C. Following incubation, a microplate spectrophotometer (Thermo Fisher Scientific, Waltham, MA, USA) with an excitation wavelength of 450 nm was used to detect absorbance. For the colony formation assay, 800 treated cells were seeded per well in a 6-well plate. After 2 weeks, colonies were fixed with methanol for 15 min and washed twice with PBS. The cells were stained with a 0.1% crystal violet solution for 10 min for further analysis. Photographs were acquired, and cell numbers were counted.

### 2.8. Migration and Invasion Assays

Migration and invasion assays were performed in 24-well plates using 8 μM pore-size Transwell filter inserts (Corning Inc., Corning, NY, USA) with or without precoated diluted Matrigel (diluted 1:8) (Corning Inc., Corning, NY, USA). Briefly, the GC cells were digested and resuspended in FBS-free DMEM or RPMI-1640 culture medium. A total of 200 μL (2.5 × 10^5^ cells/mL) of GC cells was seeded into the upper chamber, while 800 μL of complete culture medium (RPMI-1640 or DMEM with 10% FBS) was added to the lower chamber. The cells were incubated for 48 h. The infiltrated cells were counted in five random fields under a microscope (×200 magnification). Each experiment was performed in triplicate, and the mean values are shown.

### 2.9. Construction of Stable Knockdown and Overexpressed PISD Gastric Cancer Cell Line

Lentiviruses expressing shPISD (#1, #2) or shCtrl were purchased from Generalbiol (Hefei, China). LV-PISD or empty vector (PISD-NC) was purchased from Genechem (Shanghai, China). The lentiviral vectors contained all GFP reporter genes. AGS and MKN45 cells were used to establish stable PISD knockdown models, whereas MGC-803 cells were used for stable PISD overexpression experiments. Cells (3 × 10^4^) were seeded into each well and transfected with the indicated lentivirus, according to the manufacturer’s instructions. Infected cells were selected with 2 μg/mL puromycin (Sangon, Shanghai, China) for 2 weeks or more, and transfection efficiency was determined by RT-qPCR and WB analysis.

### 2.10. Subcutaneous Xenograft Experiments

All mouse experiments were approved by the Experimental Animal Ethics Committee of Anhui Medical University (Hefei, China) and conducted in accordance with the AAALAC and IACUC guidelines (approval number: LLSC20231162). Six-week-old male BALB/c nude mice were purchased from Jiangsu Jicui Yaokang Biological Co., Ltd. (Nanjing, China). AGS-shCtrl/AGS-shPISD#1 cells (1 × 10^7^), MKN45-shCtrl/MKN45-shPISD#1 cells (6 × 10^6^), and MGC-803-Vector/MGC-803-LV-PISD cells (6 × 10^6^) were separately suspended in 100 μL sterile PBS and injected subcutaneously into the left flank of nude mice (*n* = 5 per group). Tumor growth was monitored for 35 days (AGS), 7 weeks (MKN45), and 33 days (MGC-803), respectively. Tumor size in the AGS and MGC-803 models was first measured on day 7 after injection. Thereafter, tumors in the AGS and MGC-803 models were measured every 4 days. Due to the relatively slower tumor growth rate of MKN45 cells, tumor size in the MKN45 model was measured once a week. Tumor volume was calculated as 1/2 × (length × width^2^). Before reaching humane endpoints, mice were euthanized, and tumors were excised and weighed.

### 2.11. PE Content Determination

AGS cells were cultured in Petri dishes 10 cm in diameter. Mitochondria were isolated from AGS cells using a Cell Mitochondrial Isolation Kit (Beyotime, Shanghai, China) according to the manufacturer’s instructions. The mitochondrial and extramitochondrial contents were homogenized in 5% Triton X-100, and the samples were heated at 80 °C to solubilize all lipids. The mitochondrial and extramitochondrial PE content was determined using a PE assay kit (MAK361, Merck KGaA, Darmstadt, Germany). Thereafter, 10 μL of each sample (or standard) and the working reagent mix were added to each well of a 96-well plate. Fluorescence intensity was measured using an automatic microplate reader (Thermo Fisher Scientific, Waltham, MA, USA) at excitation and emission wavelengths of 535 and 587 nm, respectively.

### 2.12. Transmission Electron Microscopy

The cells were cultured in dishes 100 mm in diameter. After treatment, cells were harvested and fixed in 2.5% glutaraldehyde at 4 °C. It was then processed using the Electron Microscopy Building of Anhui Medical University. The samples were viewed under an electron microscope (Talo L120C G2, Thermo Fisher Scientific, Waltham, MA, USA) at 13,000× magnification.

### 2.13. Adenosine Triphosphate (ATP) Detection

ATP content was measured using commercial assay kits (Elabscience, Wuhan, China) according to the manufacturer’s instructions.

### 2.14. Seahorse and Cellular Metabolic Analysis

Cellular oxygen consumption rate (OCR) was measured using a Seahorse XFe24 extracellular flux analyzer (Agilent, Santa Clara, CA, USA). The cells were seeded at a density of 3 × 10^4^ cells/well into 24-well plates. Oligomycin, carbonyl cyanide 4-(trifluoromethoxy) phenylhydrazone (FCCP), and mitochondrial complex I inhibitor rotenone + mitochondrial complex III inhibitor antimycin A (Antimycin & Rotenone) were added in turn. OCR was measured under four different conditions: (1) basal levels, without any additives; (2) with oligomycin (1 μM) to inhibit ATP synthase; (3) with FCCP (1 μM), a mitochondrial uncoupler, inducing maximal respiration; and (4) with rotenone/antimycin A (0.5 μM) to end the reaction. The results were plotted using Wave software (version 2.6.1; Agilent Technologies, Santa Clara, CA, USA).

### 2.15. Mitochondrial Membrane Potential Detection

GC cells were seeded at a density of 1 × 10^5^ cells/well in 24-well plates. After cell adhesion treatment, the medium was removed by suction. Briefly, 500 µL of diluted tetramethylrhodamine ethyl ester (TMRE)-staining solution (Beyotime, Shanghai, China) was added to each well, and the mixture was incubated in a cell incubator at 37 °C for 45 min. At the end of the incubation, the cells were washed twice with preheated medium. After washing, 500 µL of medium was added to each well. They were observed under a fluorescence microscope.

### 2.16. Bioinformatic Analysis

Bioinformatic analysis was performed using web-based tools. The following public databases were searched: UALCAN database (http://ualcan.path.uab.edu, accessed on 30 September 2023) and Kaplan-Meier Plotter (http://kmplot.com/analysis/, accessed on 30 September 2023).

### 2.17. MKN45 RNA Sequencing Analysis

RNA-seq was performed in MKN45 cells with stable PISD knockdown (shPISD#1) and corresponding controls (*n* = 3 biological replicates per group). Library preparation and sequencing were conducted using Baiteng Biotechnology Co., Ltd., Beijing, China, and differential expression analysis was performed using DESeq2 with log2FC > 1 and adjusted *p* < 0.05 as thresholds.

### 2.18. Statistical Analysis

All statistical analyses were performed using GraphPad Prism version 8.0 software (GraphPad Software, San Diego, CA, USA). Data are presented as means ± standard error of the mean (SEM). The differences between the two groups were analyzed using Student’s *t*-tests; for comparisons among more than two groups, one-way or two-way ANOVA was used, followed by Tukey’s post hoc tests. Survival analyses were plotted using the Kaplan–Meier method. * *p* < 0.05, ** *p* < 0.01, and *** *p* < 0.001 indicated statistically significant differences. The ns represents no statistical significance.

## 3. Results

### 3.1. PISD Is Elevated in Gastric Cancer Tissues and Associated with Poor Prognosis

**Figure 1:**
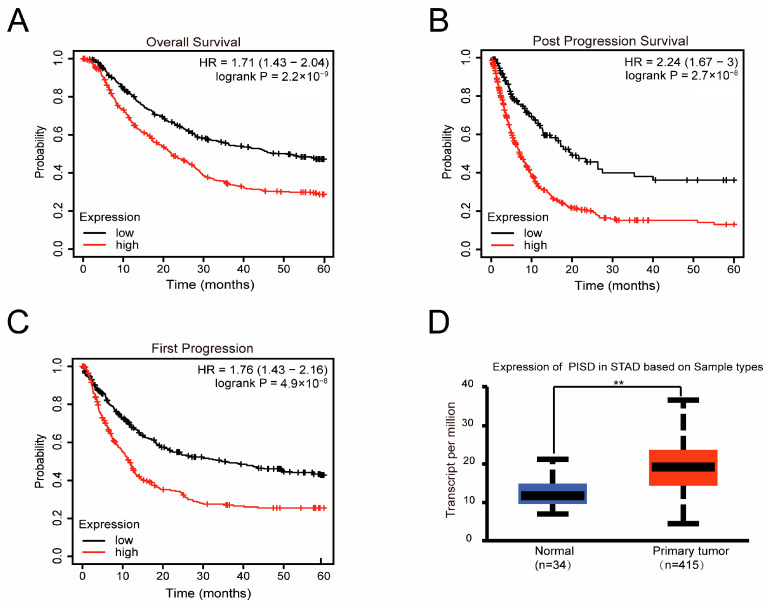
Upregulation of PISD expression in GC and its correlation with poor prognosis: (**A**–**C**) The relationship between PISD expression level and OS (**A**), PPS (**B**), and FP survival (**C**) of patients with GC was investigated using the Kaplan–Meier Plotter database. (**D**) Relative expression of PISD in normal gastric epithelial tissues (*n* = 34) and gastric cancer tissues (*n* = 415) was assessed using the UALCAN database. ** *p* < 0.01.

We analyzed the relationship between PISD expression and GC survival. Overall survival (OS), post-progression survival (PPS), and first progression (FP) were lower in patients with higher PISD levels in the Kaplan–Meier Plotter online database (Figure 1A–C). Therefore, elevated PISD expression in patients with GC indicates a poor prognosis. Moreover, PISD is a pivotal enzyme in PE synthesis; therefore, we assessed PISD expression levels in matched pairs of GC and normal tissues using the UALCAN database (Figure 1D). The analysis revealed higher PISD expression levels in GC tissues than in normal gastric epithelial tissues.

### 3.2. PISD Contributes to Malignant Phenotypes in GC Cells

**Figure 2:**
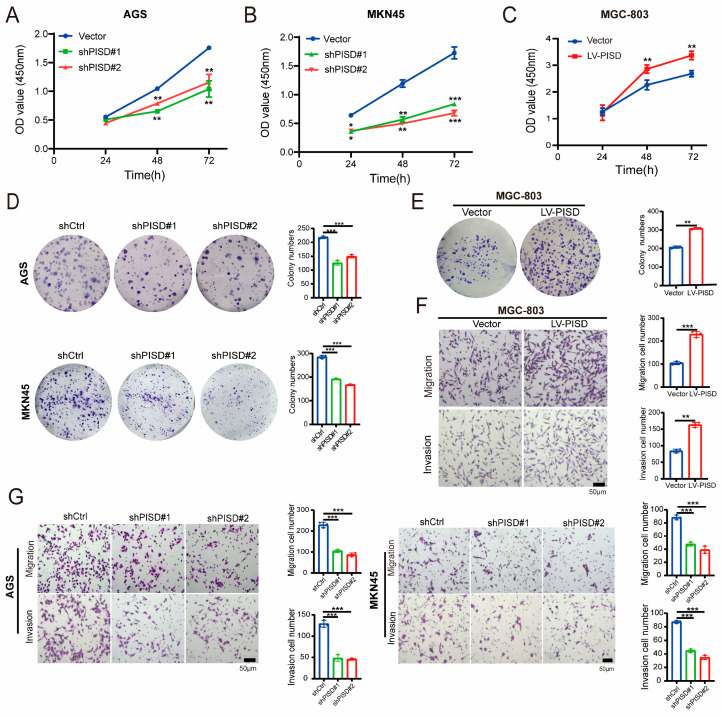
PISD contributes to the development of the malignant phenotype in GC cells: (**A**,**B**) CCK-8 assay to assess the proliferative ability of AGS and MKN45 cells following PISD downregulation. (**C**) CCK-8 assay to analyze the proliferation of stable MGC-803-Vector/MGC-803-LV-PISD cell lines. (**D**) Plate cloning experiment assessing the effect of PISD knockdown on the cloning ability of AGS and MKN45 cells. (**E**) Plate cloning experiment to assess the influence of PISD upregulation on the cloning ability of MGC-803 cells. (**F**) Transwell assays showing the effect of PISD upregulation on the migration and invasion ability of MGC-803 cells. (**G**) Transwell assay showing the effect of PISD knockdown on the migration and invasion ability of AGS and MKN45 cells. Scale bar, 50 μm. * *p* < 0.05, ** *p* < 0.01, *** *p* < 0.001.

Given the higher expression of PISD in GC samples than in normal gastric epithelial samples, the function of PISD in GC was investigated. We used AGS and MKN45 cells to silence PISD expression (Figure S1) and MGC-803 cells to overexpress PISD to assess the impact of PISD on cell behavior in vitro. The CCK-8 assay revealed that knockdown of PISD using two different PISD-shRNAs (shPISD#1 and shPISD#2) substantially attenuated the proliferation of the GC cell lines AGS and MKN45 (Figure 2A,B). In contrast, PISD overexpression promoted the proliferation of MGC-803 cells (Figure 2C). Furthermore, the plate cloning assay demonstrated that colony formation in AGS and MKN45 cells with low PISD expression was markedly inhibited (Figure 2D), whereas that in MGC-803 cells overexpressing PISD was significantly enhanced (Figure 2E).

To further evaluate the contribution of PISD to the development of migratory and invasive phenotypes in GC cells, migration and invasion assays were performed using AGS, MKN45, and MGC-803 cells. Transwell assay results indicated that PISD overexpression significantly increased the migration and invasion abilities of MGC-803 cells (Figure 2F). However, PISD knockdown significantly reduced the migration and invasion capabilities of AGS and MKN45 cells compared with their respective controls (Figure 2G). Collectively, these findings suggest that PISD is a positive regulator of the malignant behavior of GC cells.

### 3.3. PISD Expression Is Associated with Subcutaneous Xenograft Formation in Nude Mice

**Figure 3:**
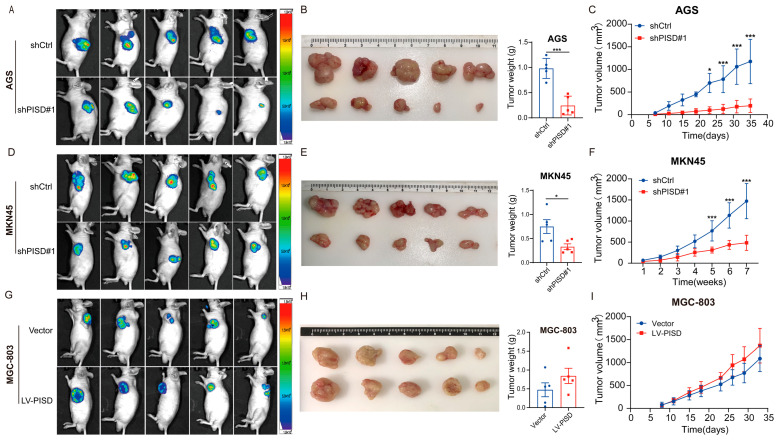
PISD expression is associated with subcutaneous xenograft formation in nude mice: (**A**–**C**) Bioluminescence imaging of tumors (**A**), dissection of tumors and tumor weight (**B**), and tumor volume (**C**) in nude mice inoculated with stable PISD-knockdown AGS cells and control cells. (**D**–**F**) Bioluminescence imaging of tumors (**D**), dissection of tumors and tumor weight (**E**), and tumor volume (**F**) in nude mice inoculated with PISD-stable knockdown MKN45 cells and control cells. (**G**–**I**) Bioluminescence imaging of tumors (**G**), dissection of tumors and tumor weight (**H**), and tumor volume (**I**) in nude mice inoculated with PISD-overexpressing MGC-803 cells and the negative control (vector group). * *p* < 0.05, *** *p* < 0.001.

To validate our in vitro findings, we further investigated the role of PISD in tumorigenicity in vivo. Stable AGS-shPISD and MKN45-shPISD cell lines with PISD knockdown, as well as MGC-803-LV-PISD cells with PISD overexpression, were established and subcutaneously injected into nude mice. Tumor growth was monitored for 35 days in the AGS model, 7 weeks in the MKN45 model, and 33 days in the MGC-803 model. Prior to euthanasia, GFP fluorescence in xenograft tumors was assessed using an in vivo imaging system. The fluorescence intensity was significantly reduced in the PISD knockdown groups compared with the corresponding control groups (Figure 3A,D). In contrast, tumors derived from PISD-overexpressing MGC-803 cells exhibited markedly increased fluorescence intensity compared with controls (Figure 3G). Consistent with the in vitro assays, knockdown of PISD markedly suppressed the tumorigenicity of AGS and MKN45 cells in nude mice, and the tumor weight in the PISD-stable knockdown group was significantly lower than that in the control group (Figure 3B,E). Conversely, overexpression of PISD promoted tumor growth in MGC-803 cells, and tumors from the PISD-overexpressing group weighed significantly more than those from the control group (Figure 3H). The volume of tumors derived from the PISD-stable knockdown group was significantly reduced compared with that of the control group (Figure 3C,F). In contrast, the tumor volume from the PISD-stable overexpression group was significantly increased (Figure 3I). Immunohistochemical staining of MGC-803 xenograft tumors showed that the LV-PISD group exhibited a higher proportion of Ki-67-positive cells compared with the vector control, indicating enhanced proliferative activity in vivo (Figure S2).

### 3.4. PISD Knockdown Results in Mitochondrial Impairment, Impacting Ferroptosis in Gastric Cancer Cells

RNA-seq analysis was performed using MKN45 gastric cancer cells with stable PISD knockdown (shPISD#1) and corresponding control cells to explore the molecular mechanisms associated with PISD depletion. Differential expression analysis identified 5748 differentially expressed genes (DEGs), including 2815 upregulated and 2933 downregulated genes (fold change > 2, adjusted *p* < 0.05). KEGG pathway enrichment analysis revealed a significant enrichment of ferroptosis-related pathways among the DEGs (Figure S3A). A volcano plot illustrated the overall distribution of DEGs, with STAT3 and GPX4 highlighted as significantly downregulated genes (Figure S3B). Upregulated genes are shown in orange, and downregulated genes are shown in blue. These results suggest that PISD knockdown is associated with alterations in ferroptosis-related signaling, potentially involving suppression of the STAT3/GPX4 axis.

**Figure 4:**
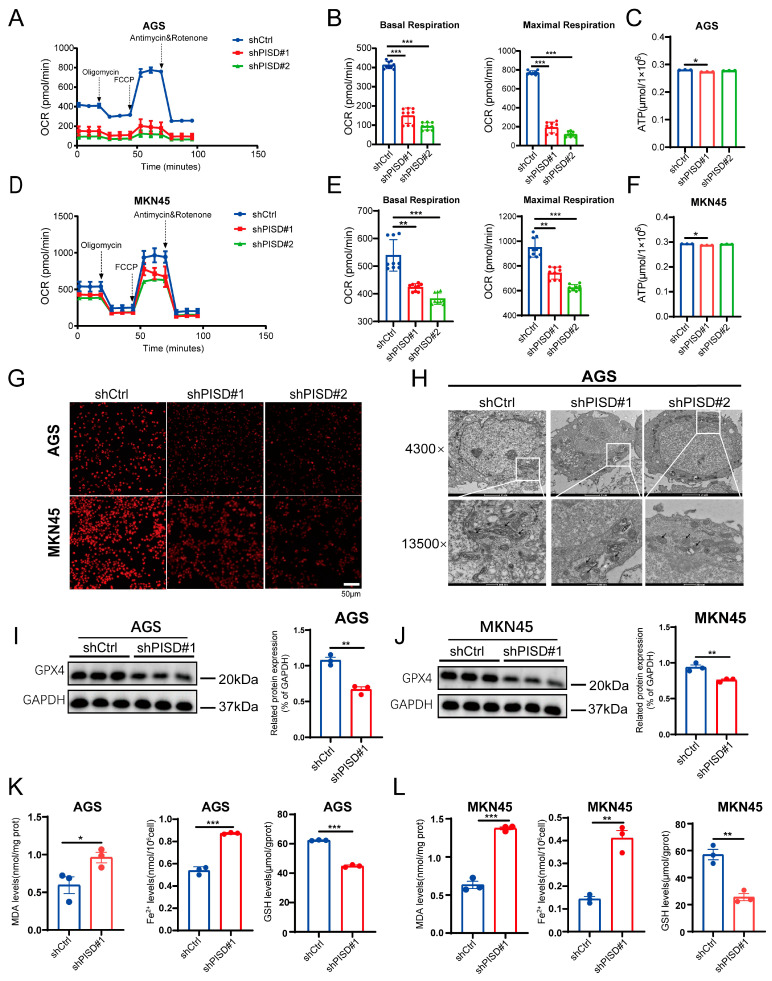
PISD knockdown inhibits mitochondrial energy metabolism and is associated with enhanced ferroptosis: (**A**) Effect of PISD knockdown on the real-time mitochondrial OCR in AGS cells. The dashed arrows indicate the time points of Oligomycin, FCCP, and Antimycin & Rotenone injection during the mitochondrial stress test. (**B**) Statistical analysis of basal and maximal levels of mitochondrial respiration in AGS cells with PISD knockdown and the control group (shCtrl group). Basal respiration was defined as the OCR prior to Oligomycin injection, and maximal respiration was defined as the OCR following FCCP injection. (**C**) Changes in the ATP content of AGS cells following PISD knockdown. (**D**) Effect of PISD knockdown on the real-time mitochondrial OCR in MKN45 cells. (**E**) Statistical analysis of the basal and maximal levels of mitochondrial respiration in MKN45 cells with PISD knockdown and the shCtrl group. (**F**) Changes in ATP content in MKN45 cells after PISD knockdown. (**G**) Detection of changes in the mitochondrial membrane potential using the TMRE probe in AGS and MKN45 cells. (**H**) Observation of ultrastructural changes in the mitochondria of AGS cells using transmission electron microscopy. (**I**,**J**) Western blotting analysis of GPX4 expression in AGS and MKN45 cells. (**K**,**L**) Quantification of MDA, Fe^2+^, and GSH levels in AGS and MKN45 cells. Scale bar, 50 μm. * *p* < 0.05, ** *p* < 0.01, *** *p* < 0.001.

Based on these initial experimental findings, we investigated the effect of PISD on GC ferroptosis. The Seahorse mitochondrial stress test was used to assess the real-time oxygen consumption rate (OCR), reflecting basal and maximal oxygen consumption during aerobic respiration. PISD knockdown led to a significant reduction in OCR in AGS cells compared to that in the negative control (Figure 4A). The essential parameters of mitochondrial function were further analyzed by evaluating OCR data at different time points. PISD knockdown notably decreased basal respiration in AGS cells, indicating reduced mitochondrial oxidative phosphorylation (Figure 4B). Furthermore, PISD knockdown significantly reduced the maximal respiration of AGS cells (Figure 4B). Similar results were observed in MKN45 cells, where PISD knockdown led to a substantial decrease in OCR (Figure 4D) and basal and maximal respiration (Figure 4E), implying that PISD knockdown inhibits mitochondrial respiration, subsequently limiting cancer cell growth.

ATP content detection showed that, compared with the control group, there were no changes in intracellular ATP levels in AGS and MKN45 cells with PISD knockdown (Figure 4C,F), similar to the results observed in MGC-803 cells overexpressing PISD (Figure S4A), suggesting that there may be a compensatory activation of the mitochondrial ATP production pathway. Additionally, mitochondrial membrane potential results showed a significant decrease in AGS and MKN45 cells following PISD knockdown compared to control cells (Figure 4G). Transmission electron microscopy revealed that mitochondria in AGS cells from the PISD-knockdown group displayed irregular mitochondrial membranes, broken cristae, and a dark matrix (Figure 4H). In contrast, the mitochondrial cristae and membranes of MGC-803 cells overexpressing PISD appeared more complete and abundant (Figure S4B).

We used PISD-knockdown AGS and MKN45 cells to examine the ferroptosis marker GPX4 and observed a marked reduction in its expression (Figure 4I,J). Ferroptosis-related indicators include malondialdehyde (MDA), ferrous iron (Fe^2+^), and glutathione (GSH). The results showed significant increases in MDA and Fe^2+^ levels, accompanied by a significant decrease in GSH levels, compared to the control group (Figure 4K,L). Collectively, these findings suggest that PISD knockdown induces mitochondrial dysfunction and is associated with enhanced ferroptosis in gastric cancer cells.

### 3.5. Downregulation of PISD Inhibits STAT3 Activation in Gastric Cancer Cells

**Figure 5:**
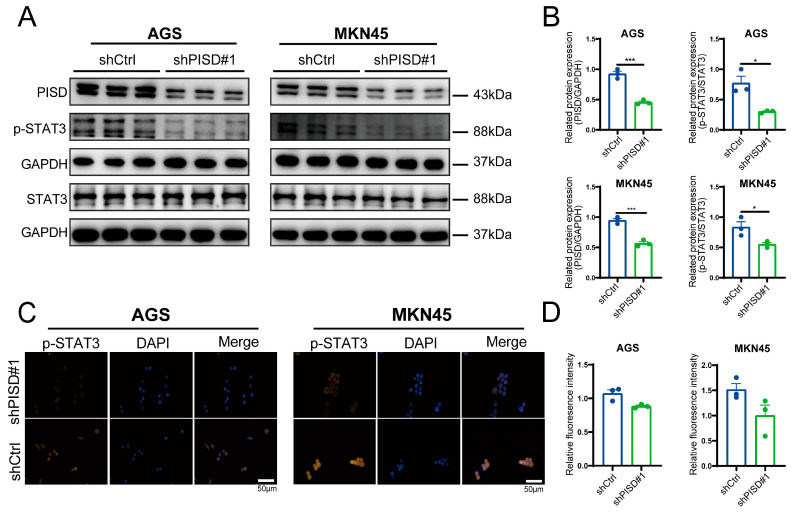
PISD knockdown suppressed STAT3 phosphorylation in gastric cancer cells: (**A**,**B**) Western blot analysis of p-STAT3 and STAT3 in AGS and MKN45 cells after PISD knockdown. (**C**,**D**) Immunofluorescence staining of p-STAT3 in AGS and MKN45 cells and quantification of fluorescence intensity. Scale bar, 50 μm. * *p* < 0.05, *** *p* < 0.001.

We performed Western blot analysis of the STAT3 signaling pathway in AGS and MKN45 cells with stable PISD knockdown. The results showed that PISD knockdown markedly reduced p-STAT3 protein levels, whereas total STAT3 expression remained unchanged or decreased slightly (Figure 5A,B). Immunofluorescence staining confirmed that PISD knockdown decreased p-STAT3 expression in both AGS and MKN45 cells (Figure 5C,D). These findings indicate that PISD downregulation is associated with reduced STAT3 phosphorylation in gastric cancer cells. In addition, we observed that both total JAK2 and phosphorylated JAK2 levels were reduced following PISD knockdown (Figure S5), suggesting that suppression of STAT3 phosphorylation may involve inhibition of upstream JAK2 signaling.

### 3.6. Supplementation with ML115 to Activate STAT3 Mitigates Ferroptosis Induced by PISD Down-Regulation in Gastric Cancer Cells

**Figure 6:**
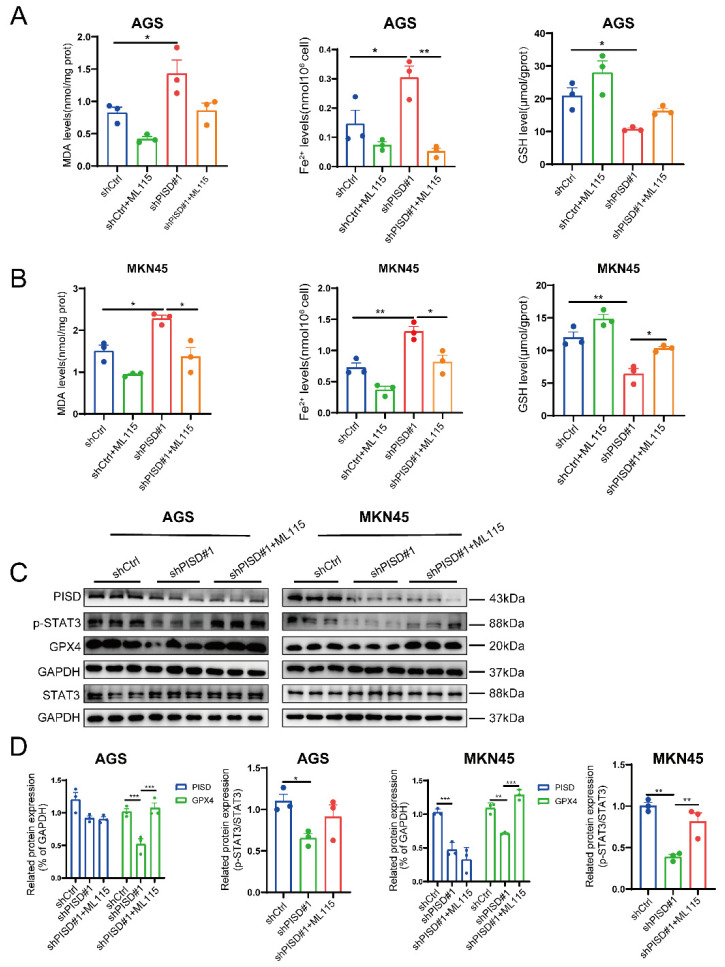
ML115 supplementation attenuated ferroptosis induced by PISD knockdown in gastric cancer cells. (**A**,**B**) Measurement of ferroptosis-related indicators (MDA, Fe^2+^, and GSH) in AGS and MKN45 cells. (**C**) Western blot analysis of PISD, STAT3, p-STAT3, and GPX4 levels in AGS and MKN45 cells receiving 20 µM ML115. (**D**) Quantification of relative protein expression levels. * *p* < 0.05, ** *p* < 0.01, *** *p* < 0.001. # represents the shRNA #2 targeting sequence.

Silencing PISD in AGS and MKN45 cells significantly increased intracellular MDA levels, promoted Fe^2+^ accumulation, and concurrently decreased cellular GSH levels. Importantly, supplementation with the STAT3 agonist ML115 markedly attenuated the accumulation of MDA and Fe^2+^ and partially restored GSH levels (Figure 6A,B), indicating a protective role of ML115 against ferroptosis triggered by PISD depletion. Western blot analysis of the STAT3/GPX4 signaling axis (Figure 6C,D) revealed that PISD knockdown led to a pronounced reduction in p-STAT3 and GPX4 protein levels, whereas ML115 supplementation increased STAT3 phosphorylation and partially restored GPX4 protein levels. Collectively, these findings support the notion that PISD silencing sensitizes gastric cancer cells to ferroptosis, accompanied by impairment of the STAT3/GPX4 pathway.

### 3.7. Fer-1 Attenuates Ferroptosis and Partially Restores Malignant Phenotypes in Gastric Cancer Cells

**Figure 7:**
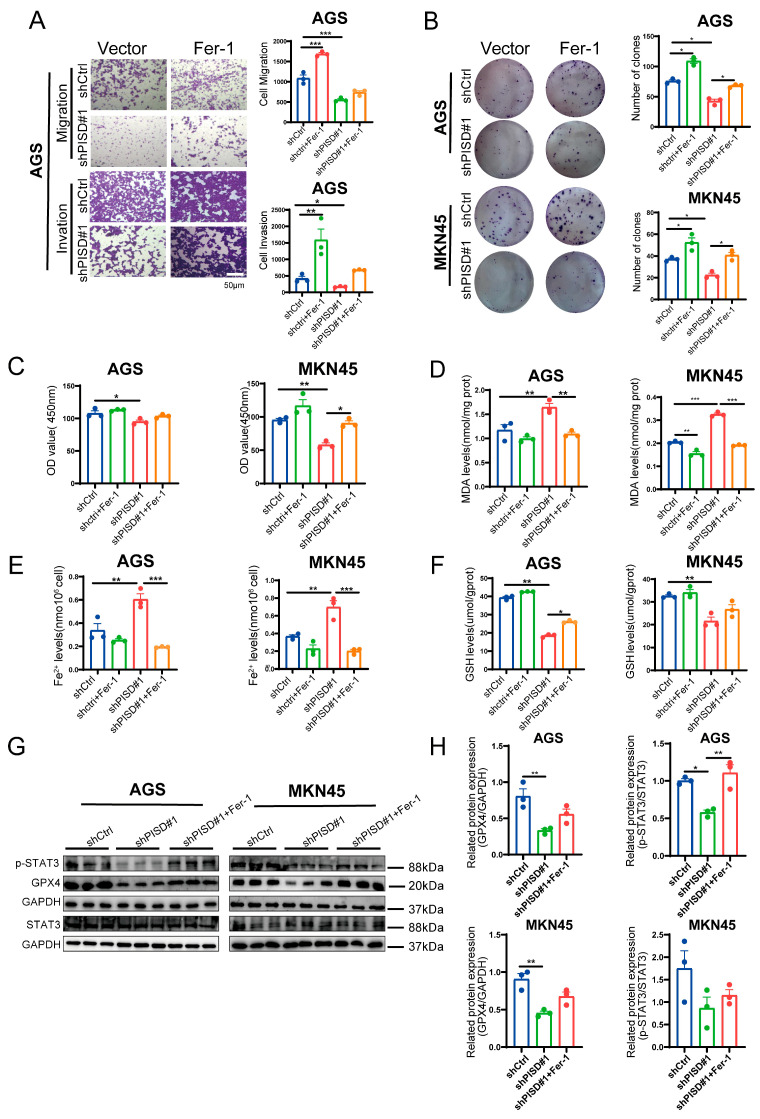
Fer-1 alleviates ferroptosis and supports the development of gastric cancer cells: (**A**) Migration and invasion assays of AGS cells with PISD knockdown and 2 μM Fer-1 treatment for 48 h. (**B**) Colony formation assays of AGS and MKN45 cells treated with 2 μM Fer-1 for 48 h. (**C–F**) Measurement of ferroptosis-related markers (MDA, Fe^2+^, and GSH) in AGS and MKN45 cells with PISD knockdown and 2 μM Fer-1 treatment. (**G**,**H**) Western blotting analysis of STAT3, p-STAT3, and GPX4 levels in AGS and MKN45 cells receiving 2 μM Fer-1. Scale bar, 50 μm. * *p* < 0.05, ** *p* < 0.01, *** *p* < 0.001.

PISD knockdown markedly suppressed the migratory and invasive abilities of AGS cells, whereas treatment with Fer-1 partially restored these phenotypes (Figure 7A). Consistently, colony formation assays revealed a significant reduction in colony number upon PISD silencing, which was substantially reversed by Fer-1 treatment (Figure 7B), further supporting the role of ferroptosis in inhibiting gastric cancer cell growth. MKN45 cell viability assays confirmed that PISD knockdown decreased proliferation, whereas Fer-1 treatment significantly rescued cell viability (Figure 7C). Silencing PISD increased MDA levels considerably (Figure 7D), elevated intracellular Fe^2+^ accumulation (Figure 7E), and reduced GSH content (Figure 7F), all hallmark features of ferroptosis. Importantly, Fer-1 treatment alleviated these alterations by suppressing MDA and iron overload while replenishing GSH, thereby attenuating ferroptotic stress (Figure 7D–F). At the signaling level, Western blot analysis demonstrated that PISD depletion reduced STAT3 phosphorylation, accompanied by downregulation of GPX4, whereas Fer-1 treatment restored STAT3 activation and GPX4 expression (Figure 7G,H). Taken together with Figure 6, these findings indicate that PISD knockdown inhibits STAT3 activation and induces ferroptosis, and that Fer-1 treatment partially restored p-STAT3 and GPX4 protein levels, thereby enhancing gastric cancer cell proliferation and survival.

### 3.8. LPE Supplementation Partially Rescues Gastric Cancer Cell Growth

**Figure 8:**
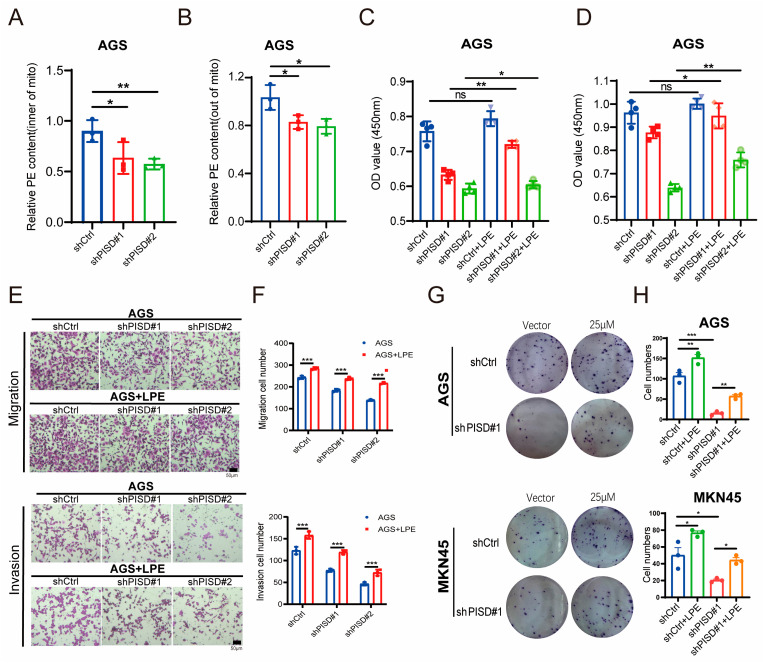
LPE supplementation rescues malignant phenotypes in PISD-deficient gastric cancer cells: (**A**,**B**) Quantification of PE levels inside and outside the mitochondria in AGS cells following PISD knockdown. (**C**,**D**) Assessment of the proliferation ability of AGS cells in each group after incubation with 25 μM LPE for 24 and 48 h using the CCK-8 assay. (**E**,**F**) Evaluation of the migration and invasion abilities of AGS cells in each group after incubation with 25 μM LPE for 48 h using Transwell migration and Matrigel invasion assays. (**G**,**H**) Colony formation assays in AGS and MKN45 cells with or without PISD knockdown and LPE supplementation (25 μM). Scale bar, 50 μm. * *p* < 0.05, ** *p* < 0.01, *** *p* < 0.001.

Given that PISD may contribute to PE synthesis in the mitochondria, the levels of PE inside and outside the mitochondria were assessed in PISD-knockdown and non-knockdown AGS cells. This revealed decreased PE levels in both the mitochondrial and cytosolic compartments of AGS cells after PISD knockdown (Figure 8A,B). Following treatment with 25 μM LPE for 24 and 48 h, the CCK-8 assay demonstrated that LPE supplementation promoted the growth of AGS cells in the PISD-knockdown group (Figure 8C,D). Furthermore, the migration and invasion assay results indicated that LPE supplementation increased the migration and invasion abilities of AGS cells with PISD knockdown (Figure 8E,F). Similarly, colony formation assays showed that the loss of PISD dramatically reduced clonogenic growth of both AGS and MKN45 cells, whereas LPE supplementation significantly rescued colony formation (Figure 8G,H).

### 3.9. LPE Supplementation Alleviates Ferroptosis in Gastric Cancer Cells

**Figure 9:**
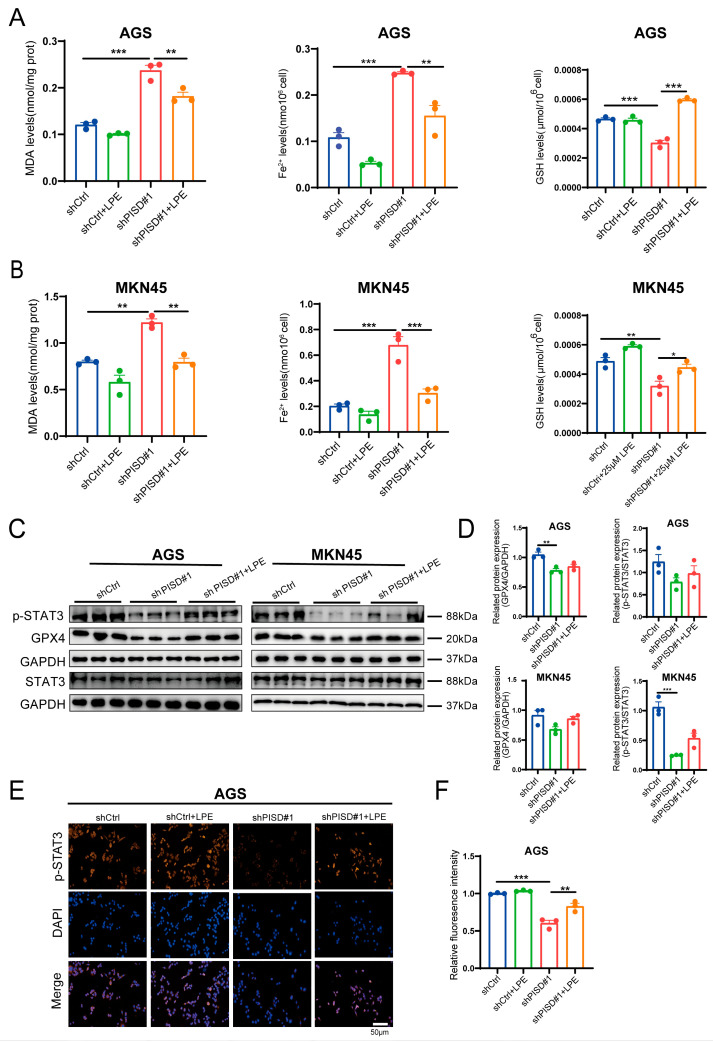
LPE supplementation alleviates ferroptosis by reactivating the STAT3/GPX4 signaling pathway in gastric cancer cells: (**A**,**B**) Measurement of ferroptosis-related indicators (MDA, Fe^2+^, and GSH) in AGS and MKN45 cells with 25 μM LPE for 48 h. (**C**,**D**) Western blot analysis of STAT3, p-STAT3, and GPX4 signaling proteins receiving 25 μM LPE. (**E**,**F**) Expression of p-STAT3 in AGS cells was determined by immunofluorescence staining after incubation with 25 μM LPE for 48 h. Scale bar, 50 μm. * *p* < 0.05, ** *p* < 0.01, *** *p* < 0.001.

PISD knockdown markedly increased MDA and intracellular Fe^2+^ levels and decreased GSH levels, indicating enhanced lipid peroxidation and ferroptosis. Treatment with 25 μM LPE effectively reversed these changes and restored redox balance (Figure 9A,B). Western blot analysis further demonstrated that PISD knockdown reduced the protein levels of p-STAT3 and GPX4, whereas LPE treatment partially rescued their expression (Figure 9C,D). Immunofluorescence assays showed that PISD knockdown significantly diminished nuclear p-STAT3 signaling in AGS cells, which was partially restored by LPE supplementation (Figure 9E,F). Collectively, these results indicate that LPE supplementation alleviated ferroptosis and was accompanied by a partial restoration of STAT3 phosphorylation.

### 3.10. Proposed Mechanistic Model of PISD Downregulation 

**Figure 10:**
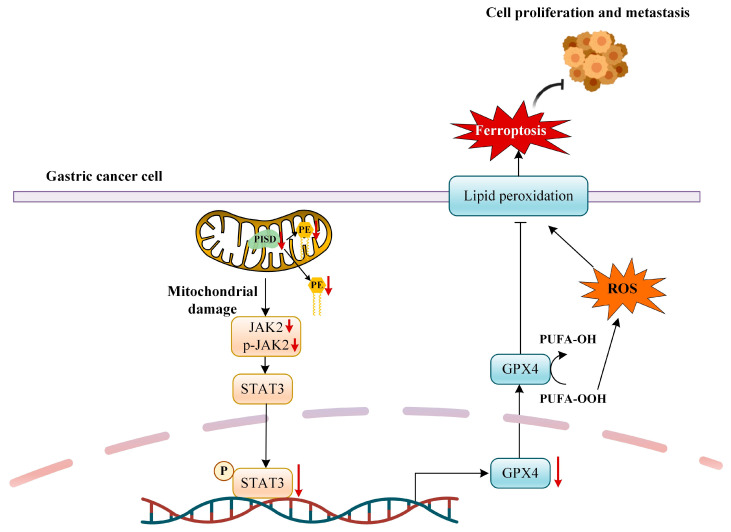
Proposed model illustrating how PISD downregulation is associated with mitochondrial dysfunction and ferroptosis in gastric cancer.

Based on these in vitro and in vivo findings, we propose a working model illustrating how PISD downregulation induces mitochondrial dysfunction and ferroptosis in gastric cancer cells (Figure 10). PISD downregulation disrupts mitochondrial PE homeostasis in both inner and outer membranes, leading to mitochondrial dysfunction. PISD depletion is associated with reduced JAK2 abundance and phosphorylation, accompanied by decreased p-STAT3. Suppression of the JAK2/STAT3 axis contributes to reduced GPX4 expression, thereby promoting ROS accumulation, lipid peroxidation, and ferroptosis, ultimately inhibiting gastric cancer cell proliferation and metastasis. Solid arrows indicate activation or promotion, whereas blunt-ended lines indicate inhibition. Red downward arrows represent downregulation of molecules or pathways. The dashed line represents the boundary between the cytoplasm and the nucleus.

## 4. Discussion

Despite remarkable progress in GC management in recent decades [30,31], chemotherapy resistance and severe systemic toxicities remain major obstacles to improving patient outcomes [32]. Hence, the development of novel therapeutic strategies that can selectively eliminate tumor cells with minimal toxicity is urgently required. Ferroptosis is an iron-dependent, lipid-peroxidation-driven form of regulated cell death that has emerged as a promising approach in cancer therapy [33,34,35,36,37]. As a key mitochondrial enzyme that catalyzes the conversion of phosphatidylserine to PE, PISD plays a vital role in maintaining mitochondrial phospholipid homeostasis and bioenergetic functions. However, its functional significance in GC and ferroptosis remains largely unknown.

In the present study, we demonstrated that PISD downregulation suppressed gastric tumor progression by promoting ferroptosis. Transcriptomic and Kyoto Encyclopedia of Genes and Genomes pathway analyses identified ferroptosis as one of the principal pathways associated with PISD-mediated cell death. Functionally, PISD knockdown resulted in impaired mitochondrial respiration, as evidenced by a reduced OCR, decreased membrane potential, and disrupted mitochondrial morphology. These findings indicate that PISD is indispensable for mitochondrial integrity and that its depletion leads to metabolic stress that predisposes GC cells to ferroptotic death. While our results strongly support ferroptosis as the primary mechanism of PISD depletion-induced cell death, we acknowledge that other cell death pathways, such as apoptosis (e.g., caspase activation, Annexin V/PI staining) or necroptosis (e.g., MLKL phosphorylation), may also contribute to PISD depletion-induced cell death. However, due to time and scope limitations, these pathways were not comprehensively evaluated in the current study. Future research will expand the evaluation of additional cell death pathways to further elucidate their contributions to PISD depletion-induced cell death.

Notably, although PISD knockdown markedly diminished mitochondrial respiration, intracellular ATP levels remained unchanged in both AGS and MKN45 cells; similar results were observed in PISD-overexpressing MGC-803 cells. This suggests that cancer cells may compensate for impaired oxidative phosphorylation by upregulating extra-mitochondrial ATP-generating pathways, such as glycolysis. This metabolic compensation aligns with the concept of cancer metabolic plasticity, allowing tumor cells to preserve energy homeostasis despite mitochondrial dysfunction. Therefore, while PISD depletion compromises mitochondrial function, it may also induce adaptive metabolic reprogramming, which influences sensitivity to ferroptosis. Mechanistically, PISD knockdown altered ferroptosis-related biochemical parameters, including elevated levels of MDA and Fe^2+^ and decreased GSH and GPX4 expression. The ferroptosis inhibitor Fer-1, which is specifically accompanied by a reduction in GPX4-dependent lipid peroxidation [38], effectively rescued PISD downregulation-induced cell death and redox imbalance.

Furthermore, the inhibition of STAT3 phosphorylation was accompanied by a marked increase in intracellular Fe^2+^ levels. STAT3 signaling is closely linked to iron homeostasis, particularly through IL-6/STAT3-dependent induction of hepcidin (HAMP), a central regulator of systemic and cellular iron balance [39]. Hepcidin binds to the iron exporter ferroportin (FPN1/SLC40A1) and promotes its internalization and degradation, thereby limiting iron export and favoring intracellular iron retention [40]. Altered iron retention can subsequently reshape iron uptake and storage programs, including transferrin receptor 1 (TfR1)-mediated iron uptake and ferritin-based iron sequestration, in a context-dependent manner [41]. In our study, PISD depletion reduced p-STAT3 and was accompanied by increased labile Fe^2+^ levels, suggesting that impaired STAT3 signaling may contribute to iron dysregulation. The expanded Fe^2+^ pool can promote Fenton reactions, generating hydroxyl radicals and amplifying lipid peroxidation, thereby sensitizing gastric cancer cells to ferroptotic stress. Notably, treatment with the ferroptosis inhibitor Fer-1 not only suppressed lipid peroxidation but also alleviated intracellular Fe^2+^ overload. One possible explanation is that Fer-1 acts as a radical-trapping antioxidant, scavenging lipid peroxyl radicals and interrupting iron-dependent lipid peroxidation chain reactions, thereby attenuating ferroptotic oxidative damage. In addition, Fer-1 treatment was associated with restoration of STAT3 phosphorylation and GPX4 expression in our experimental system, suggesting a potential link between ferroptosis inhibition and STAT3/GPX4 signaling. Collectively, these results suggest that p-STAT3 acts as a pivotal regulator of intracellular iron balance during ferroptosis and that pharmacological inhibition of ferroptosis can mitigate Fe^2+^ accumulation by reactivating the STAT3/GPX4 axis.

Given the central role of GPX4 in neutralizing lipid peroxides, the downregulation is a hallmark of ferroptosis [42]. Signal transducer and activator of STAT3, a transcription factor closely linked to ferroptosis regulation [43,44], has been identified as a key downstream target. Prior studies have reported that the pharmacological inhibition of SREBP1 is accompanied by a reduction in pancreatic cancer growth via inducing GPX4-mediated ferroptosis [45] and that polyphyllin VI triggers ferroptosis by inhibiting STAT3 phosphorylation in hepatocellular carcinoma [46]. We observed that PISD depletion significantly reduced GPX4 protein levels, given that STAT3 functions as a transcription factor known to regulate GPX4 expression. However, our study has not yet explored in detail the effects of PISD depletion on STAT3 localization or its transcriptional activity. These aspects will be explored as future directions for further research. In addition, we observed that both total JAK2 and phosphorylated JAK2 were reduced following PISD depletion, suggesting that impaired STAT3 activation may involve suppression of upstream JAK2 signaling. However, whether JAK2 may directly contribute to the link between mitochondrial lipid metabolism and STAT3 activation requires further investigation. Our data suggest that PISD may regulate ferroptosis through the JAK2/STAT3/GPX4 signaling cascade.

Collectively, our results reveal a previously unrecognized role of PISD in regulating ferroptosis in gastric cancer. PISD depletion disrupts mitochondrial phosphatidylethanolamine homeostasis and impairs mitochondrial function, which is accompanied by reduced JAK2 abundance and phosphorylation, decreased STAT3 activation, and downregulation of GPX4 expression. As GPX4 is a central antioxidant enzyme that detoxifies lipid peroxides, its reduction may contribute to lipid peroxidation and ferroptotic cell death. Importantly, activation of STAT3 using ML115 partially restored GPX4 protein levels and alleviated ferroptosis-related phenotypes, supporting the involvement of the JAK2/STAT3/GPX4 axis in this process. Future studies are warranted to determine the precise molecular mechanisms by which mitochondrial lipid metabolic alterations regulate JAK2/STAT3 signaling and to clarify whether GPX4 is regulated transcriptionally or post-transcriptionally downstream of STAT3.

## 5. Conclusions

In conclusion, our study highlights the critical role of PISD in GC progression by regulating PE synthesis and mitochondrial function. PISD downregulation reduces PE levels in and outside mitochondria, disrupts mitochondrial energy metabolism, and facilitates ferroptosis in GC cells. Mechanistically, PISD depletion is associated with reduced JAK2/STAT3 signaling and decreased GPX4 expression, which may contribute to enhanced ferroptosis. Together, our findings support a working model in which PISD depletion promotes ferroptosis through mitochondrial dysfunction and suppression of the JAK2/STAT3/GPX4 signaling axis in gastric cancer cells.

## Data Availability

The RNA-seq data generated in this study and other raw data supporting the conclusions of this article are available from the corresponding author upon reasonable request.

## Supplementary Materials

The following supporting information can be downloaded at https://www.mdpi.com/article/10.3390/cimb48030300/s1.
