# Correction: Wang et al. Phosphatidylserine Decarboxylase Promotes Ferroptosis Through STAT3/GPX4 Signaling in Gastric Cancer. *Curr. Issues Mol. Biol.* 2026, *48*, 300

**DOI:** 10.3390/cimb48060590

**Published:** 2026-06-03

**Authors:** Li Wang, Yaoxing Wang, Mingkai Shao, Tao Wang, Wanbao Zheng, Jun Cao, Renwen Luo, Youyan Tu, Yiting Xia, Yiming Wei, Ning Liu, Wenjie Lu, Youzhi Xu

**Affiliations:** 1 College of Basic Medicine, Anhui Medical University, Hefei 230032, China; 2345010251@stu.ahmu.edu.cn (L.W.); wyx18473449151@126.com (Y.W.); 2345010097@stu.ahmu.edu.cn (M.S.); 2313100046@stu.ahmu.edu.cn (Y.X.); w18726870301@163.com (Y.W.); 2 Inspection Department, Yuetang District Center for Disease Control and Prevention, Xiangtan 411100, China; 3 School of Pharmacy, Anhui Medical University, Hefei 230032, China; 2345010146@stu.ahmu.edu.cn (T.W.); 2345010176@stu.ahmu.edu.cn (W.Z.); caojun20201314@163.com (J.C.); 2445010199@stu.ahmu.edu.cn (R.L.); 2445010150@stu.ahmu.edu.cn (Y.T.); liunky@163.com (N.L.); 4 School of Life Sciences, Westlake University, Hangzhou 310024, China

## Error in Figure

In the original publication [1], during figure assembly, the GAPDH loading control band for STAT3 in the AGS cell panel was inadvertently taken from the membrane image that served as the GAPDH control for p-STAT3, rather than using its own independent GAPDH image from a separate experiment.

This error occurred only during figure preparation; the raw data and all experimental results remain entirely correct and unchanged. No conclusions of the paper are affected.

The correction has been made by replacing the GAPDH band for STAT3 in Figure 6 with the correct, independent GAPDH band from the original experiment. The corrected Figure 6 appears below. The authors state that the scientific conclusions are unaffected. This correction was approved by the Academic Editor. The original publication has also been updated.

## Figures and Tables

**Figure 6:**
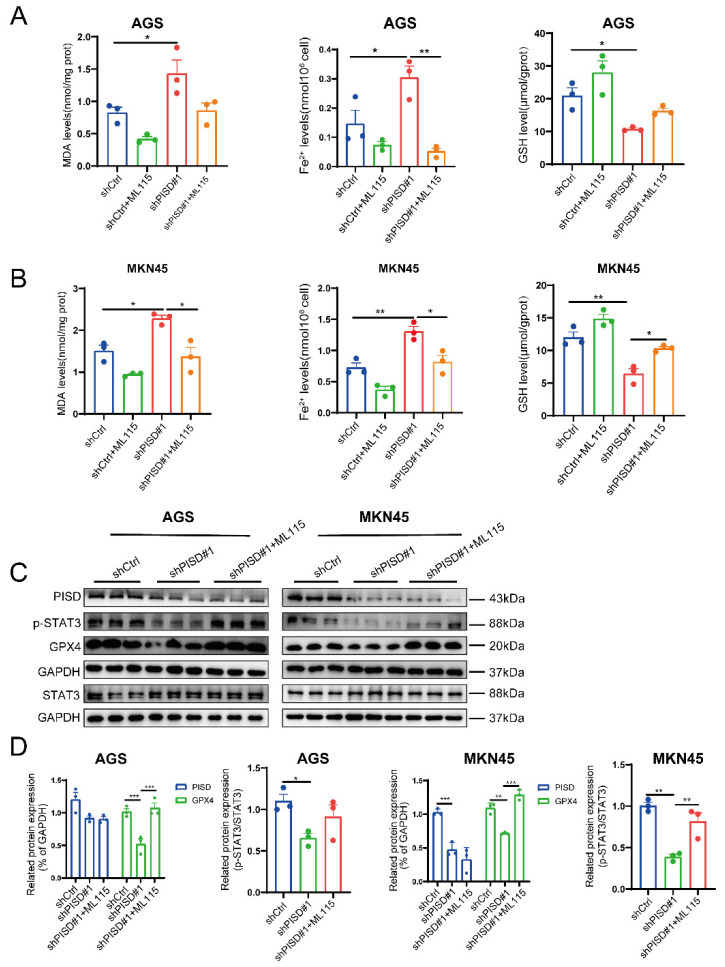
ML115 supplementation attenuated ferroptosis induced by PISD knockdown in gastric cancer cells. (**A**,**B**) Measurement of ferroptosis-related indicators (MDA, Fe^2+^, and GSH) in AGS and MKN45 cells. (**C**) Western blot analysis of PISD, STAT3, p-STAT3, and GPX4 levels in AGS and MKN45 cells receiving 20 µM ML115. (**D**) Quantification of relative protein expression levels. * *p* < 0.05, ** *p* < 0.01, *** *p* < 0.001. # represents the shRNA #2 targeting sequence.
