# Supplementary material for: Phosphatidylserine Decarboxylase Promotes Ferroptosis Through STAT3/GPX4 Signaling in Gastric Cancer
Source: Curr Issues Mol Biol. 2026 Mar 11;48(3):300. doi: 10.3390/cimb48030300 (PMC13025512; doi:10.3390/cimb48030300)
Supplement: Supplementary file 1 [file cimb-48-00300-s001.zip › cimb-4156772-supplementary.pdf]

Supplementary Information

PISD Promotes Ferroptosis through STAT3/GPX4 Signaling in Gastric Cancer

Li Wang[1,#], Yaoxing Wang[1,3,#], Mingkai Shao[1], Tao Wang[2], Wanbao Zheng[2], Jun Cao[2], Renwen Luo[2], Youyan Tu[2], Yiting Xia[1], Yiming Wei[1], Ning Liu[2,4], Wenjie Lu[2,*], Youzhi Xu[1,*],

[1]College of Basic Medicine, Anhui Medical University, Hefei, Anhui, 230032, China

[2]School of Pharmacy, Anhui Medical University, Hefei, Anhui, 230032, China

[3]Inspection Department, Yuetang district Center for Disease control and Prevention, Xiangtan, Hunan, 411100, China

[4]School of Life Sciences, Westlake University, Hangzhou, 310000, China

[#] These authors contributed equally to this work.

*Corresponding authors: Youzhi Xu (xuyouzhi@ahmu.edu.cn, ORCID: 0000-0003-2160-7576, Tel: +86-551-65161129), Wenjie Lu (wenjielu@ahmu.edu.cn, ORCID: 0000-0003-1741-2311, Tel: +86-551-65161133).

Supplementary figures and figure legends

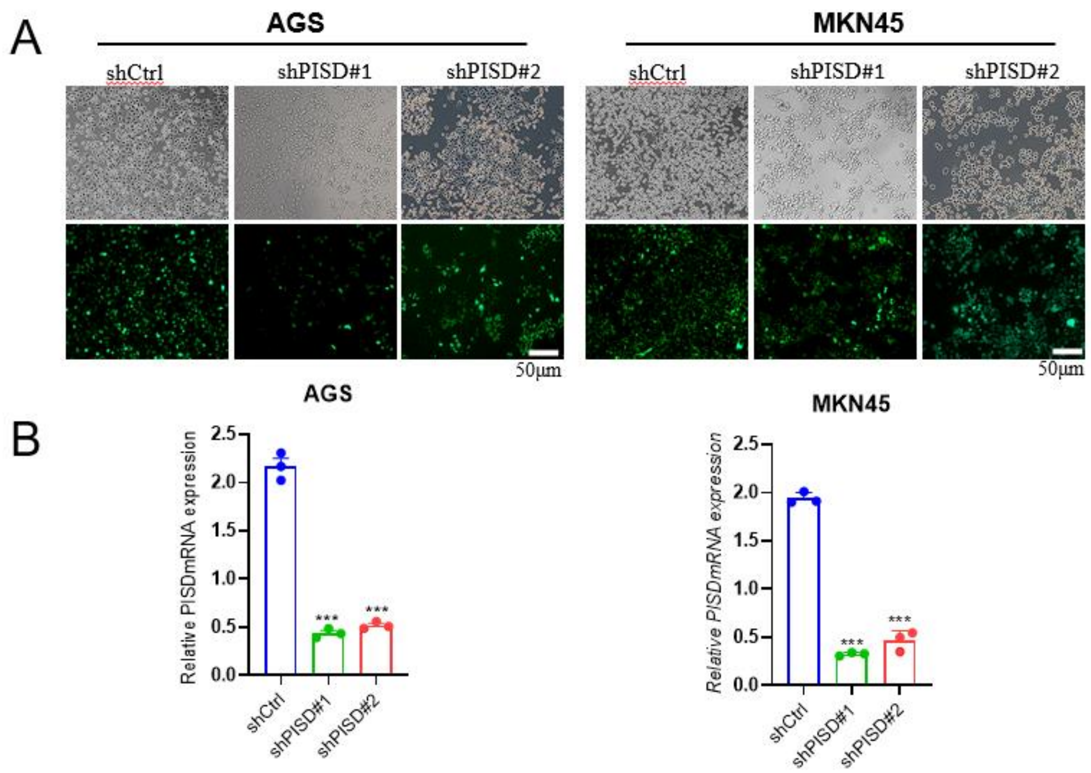

Figure S1. Effects of PISD modulation on the behavior of gastric cancer cells. (A) Transfection efficiency following PISD knockdown, assessed using fluorescence microscopy (100×). (B) qRT-PCR analysis of PISD expression following knockdown. Scale bar, 50 μm. Data are presented as mean ± SEM.

A

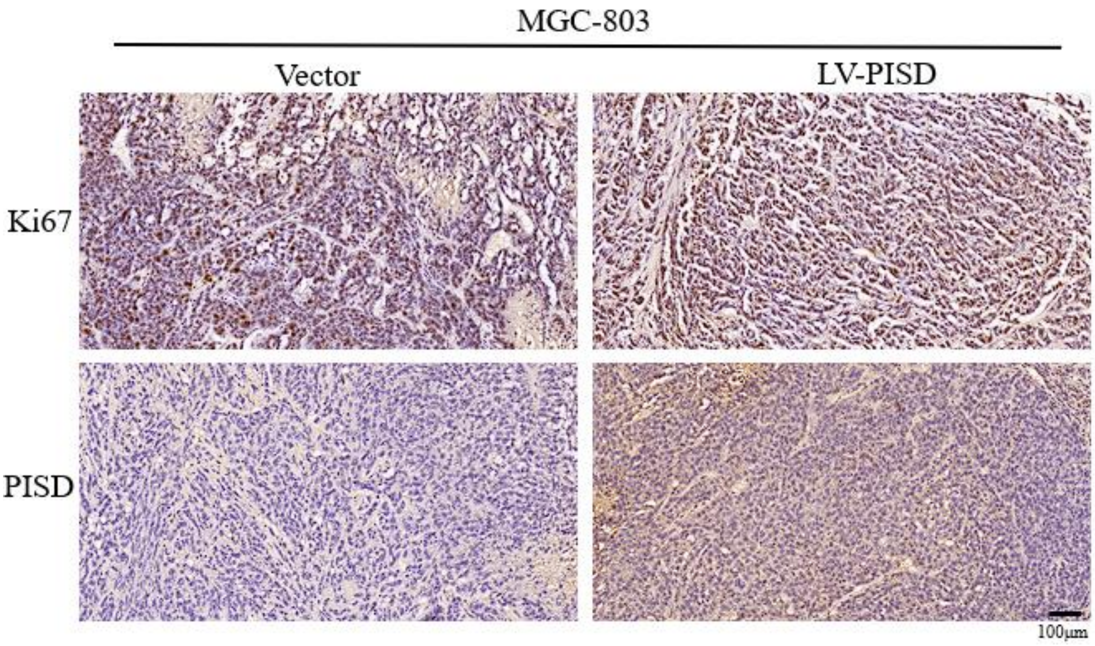

Figure S2. In vivo validation of the role of PISD in gastric cancer progression. (A) Immunohistochemical staining for Ki-67 in tumor tissues from xenograft mouse models, used to assess proliferative activity. Scale bar,50 μm.

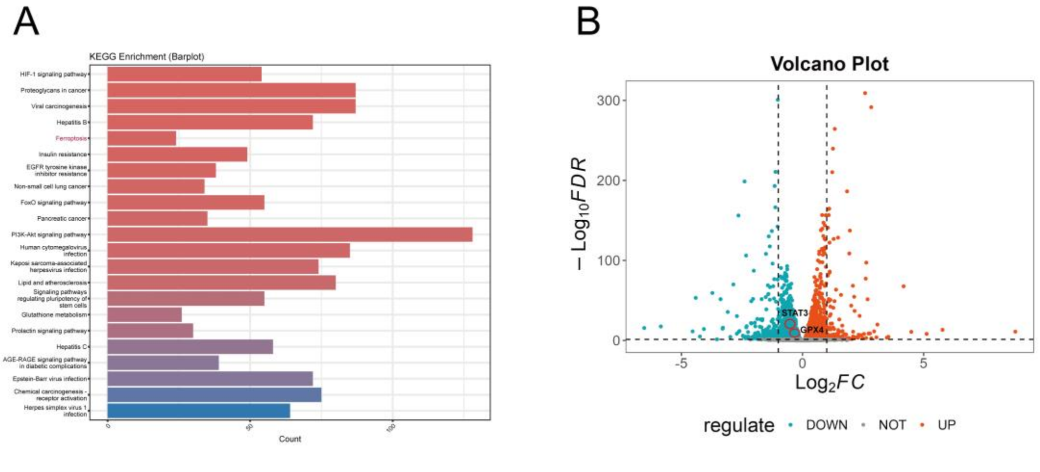

Figure S3. Transcriptomic analysis of PISD knockdown in GC cells. (A) KEGG pathway enrichment analysis of differentially expressed genes (DEGs). (B) Volcano plot showing DEGs between PISD knockdown and control groups. STAT3 and GPX4 were significantly downregulated (fold change > 2, $P < 0.05$).

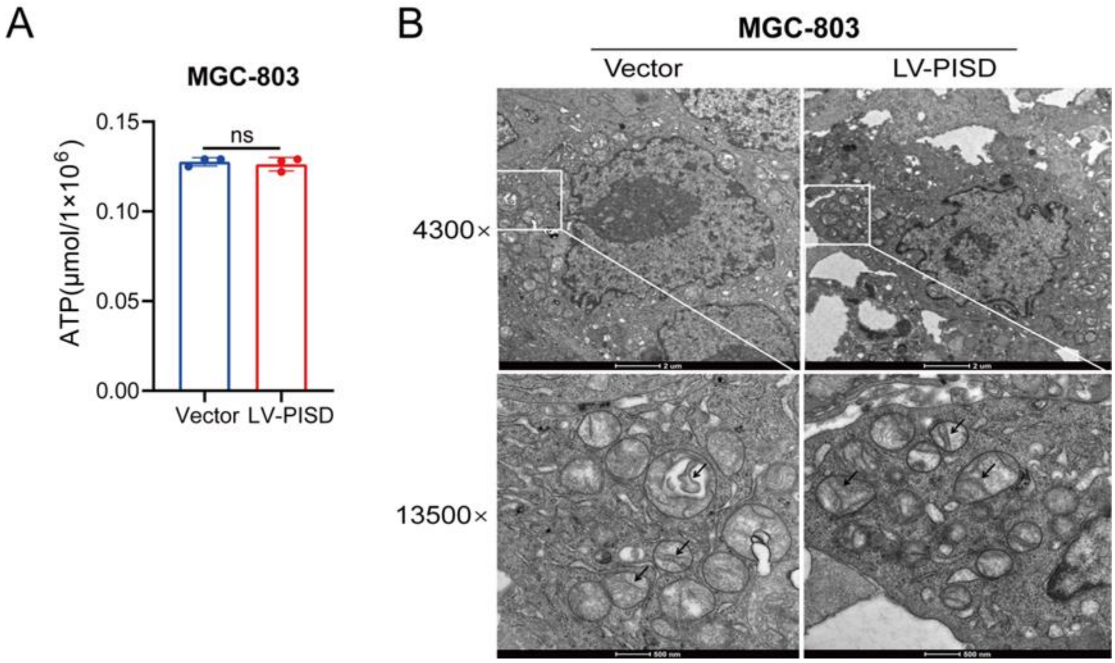

Figure S4. Effects of PISD overexpression on mitochondrial function. (A) ATP content in MGC-803 cells following PISD overexpression. (B) Transmission electron microscopy images showing mitochondrial ultrastructure in the PISD-overexpression group and vector control group. Scale bar, 500 nm. Data are presented as mean ± SEM.

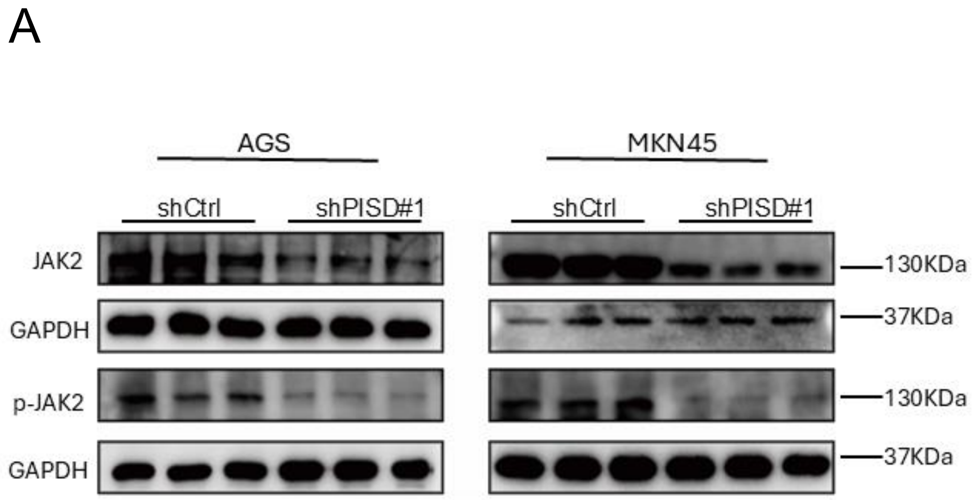

Figure S5. PISD knockdown suppressed JAK2 and p-JAK2 in gastric cancer cells. (A) Western blot analysis of JAK2 and p-JAK2 in AGS and MKN45 cells after PISD knockdown.
